# Future opportunities for the Athlete Biological Passport

**DOI:** 10.3389/fspor.2022.986875

**Published:** 2022-11-02

**Authors:** Bastien Krumm, Francesco Botrè, Jonas J. Saugy, Raphael Faiss

**Affiliations:** ^1^Research and Expertise in Anti-Doping Sciences, Institute of Sport Sciences, University of Lausanne, Lausanne, Switzerland; ^2^Laboratorio Antidoping, Federazione Medico Sportiva Italiana, Rome, Italy

**Keywords:** anti-doping, Athlete Biological Passport, blood, urine, serum, biomarkers

## Abstract

The Athlete Biological Passport (ABP) was introduced to complement the direct anti-doping approach by indirectly outlining the possible use of prohibited substances or methods in sports. The ABP proved its effectiveness, at least through a deterrent effect, even though the matrices used for longitudinal monitoring (urine and blood) are subject to many intrinsic (e.g., genetic) and extrinsic (e.g., environmental conditions) confounding factors. In that context, new and more specific biomarkers are currently under development to enhance both the sensitivity and the specificity of the ABP. Multiple strategies are presently being explored to improve this longitudinal monitoring, with the development of the current modules, the investigation of new strategies, or the screening of new types of doping. Nevertheless, due to the variability induced by indirect biomarkers, the consideration of confounding factors should continuously support this research. Beyond tremendous advances in analytical sensitivity, machine learning-based approaches seem inevitable to facilitate an expert interpretation of numerous biological profiles and promote anti-doping efforts. This perspective article highlights the current innovations of the Athlete Biological Passport that seem the most promising. Through different research axes, this short manuscript provides an opportunity to bring together approaches that are more widely exploited (e.g., omics strategies) and others in the early stages of investigation (e.g., artificial intelligence) seeking to develop the ABP.

## Introduction

The Athlete Biological Passport (ABP) was first introduced in 2009 to thwart doping practices in sports by indirectly pointing out the possible use of prohibited substances or methods. Through longitudinal, individual, and adaptive monitoring, the ABP was also developed to target athletes requiring special attention (1) for the subsequent direct detection of prohibited substances in their urine or blood. While laboratory detection techniques have improved massively (2), the short detection window for some substances remains the major limitation to direct detection (3). The first ABP module implemented includes hematological markers sensitive to different blood doping protocols, such as different types of erythropoiesis-stimulating agents (ESA) or autologous blood transfusions (4). A second module was developed to deal with doping by the so-called “pseudo-endogenous steroids” (that are, endogenous steroids when administered exogenously), with a screening of urinary biomarkers (1). Based on priors using a Bayesian network (5), the threshold values for each athlete are individualized as measurement points are recorded (4). With more than 180 Anti-Doping Rule Violations (ADRV) since its implementation, the ABP proved its effectiveness, at least through a deterrent effect outlined by a putative reduced amplitude of doping practices (6).

**Figure 1:**
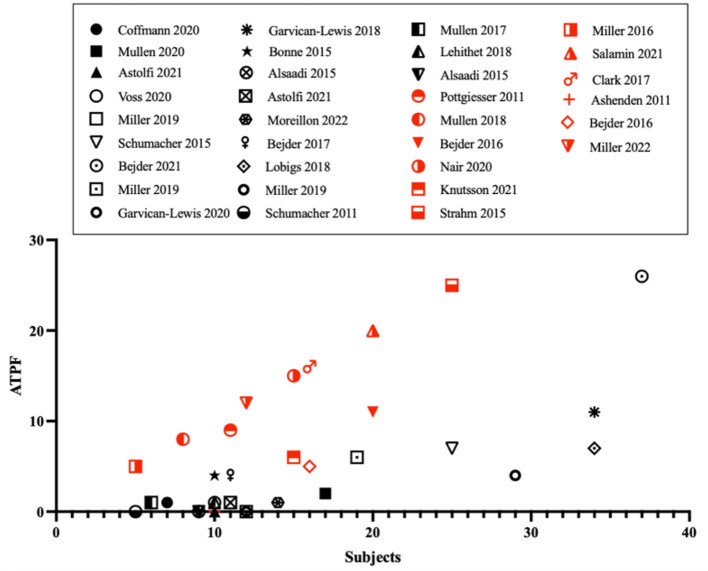
Occurrences of atypical passport findings (ATPF) as function of the number of subjects in the studies that used the official Anti-Doping Administration and Management System (ADAMS) training software. Publications involving doping protocols are presented with red symbols, publications investigating confounding factors in black figures.

The confounding factors impacting the current variables of the ABP remain one of the main limitations of the indirect approach. For the hematological module, the impact of several factors on plasma volume (e.g., physical exercise) and erythropoiesis (e.g., altitude training) have been identified (7). Further, the influence of exogenous (e.g., alcohol) and endogenous factors (e.g., menstrual cycle) were shown to alter variables of the steroid module (8). We have performed a comparative analysis of studies published in peer-reviewed journals, and reporting ABP profiles from the Anti-Doping Administration and Management System (ADAMS). Very interestingly, despite a higher occurrence of Atypical Passport Findings (ATPF) in protocols involving prohibited substances, atypical profiles were also found in connection with several physiological confounders (Figure 1). Combined with a decrease in sensitivity when micro-dosing treatment is implemented (9), the development of new specific biomarkers is required. In this context, this perspective article aims to highlight promising approaches for the future development of the ABP.

## Development of current modules

Variables analyzed by flow cytometry (with the Sysmex XN instruments generation since 2019) are now used for the measurement of the ABP hematological variables (10). Using a slightly modified technology for reticulocytes measurement compared to the previous XE and XT series (11), this latest generation of devices provides the quantification of many blood parameters currently not yet exploited by the ABP. For instance, reticulocyte hemoglobin equivalent (Ret-He) or immature reticulocyte fraction (IRF) could be potential candidates for the development of the ABP hematological module (12). These latter variables are indeed responsive to erythropoiesis alteration and independent of acute PV shift (13). Considered as a potential marker of iron deficiency (14), Ret-He could besides contribute to the existing work related to iron metabolism in the ABP framework (15–17). In addition, microvolumetric capillary whole blood collections have recently been suggested as an alternative to venous blood sampling (18). Demonstrating excellent agreement when generating individual ABP profiles, this collection method could become particularly useful in sample collection and need to be further explored.

Research seeking to identify new biomarkers to discriminate doping practices is the most investigated approach. However, the development of markers to discriminate the effects of confounding factors is a further alternative. In this way, the development of corrected individual limits by combining multiple markers sensitive to plasma volume (PV) variations has been suggested (19). This model was further validated in elite athletes, allowing them to discern hemoglobin concentration ([Hb]) changes caused by an alteration of erythropoiesis from those resulting from a transient hemodilution caused by physical exercise (20) or altitude exposure (21). The validity of these latter serum biomarkers in assessing PV variations was additionally shown in women monitored over 8 weeks (22). The model, therefore, seems particularly promising in encompassing all forms of natural variations in PV over time and could be considered useful in the toolbox for the interpretation of individual ABP profiles.

To complement the current steroid module, the development of a “blood steroid profile” is currently being developed (23, 24). Through ultra-high performance liquid chromatography-high-resolution mass spectrometry (UHPLC-HRMS) analysis, a method to quantify several endogenous steroids in serum has notably been implemented (23). Subsequently, a longitudinal evaluation of multiple serum biomarkers following the administration of transdermal testosterone (T) in women was performed, where the T/androstenedione ratio demonstrated the higher sensitivity during treatment (24). While a recent study reports an urgent need to develop biomarkers specific to women (25), serum monitoring has already demonstrated the potential of serum T measurement by liquid chromatography-mass spectrometry (LC-MS) to detect T doping in female athletes (26).

The isotope ratio mass spectrometry (IRMS) test is currently performed in case of suspicious variations of a steroid profile to confirm the non-endogenous origin of steroids (27). However, detecting abnormal isotopic values in steroid profile not identified as suspicious by the current criteria of the steroidal module of the ABP, longitudinal IRMS has shown a better sensitivity (28) and a lower individual variability (29, 30) in comparison to the traditional approach, based on the concentrations and concentration ratios of the steroid markers. Moreover, as the confirmation procedure is usually costly and time-consuming, a fast IRMS analysis has demonstrated adequate selectivity providing the examination of a larger number of samples (31). Longitudinal monitoring of 13C values of the target steroids and of the endogenous reference compounds would for instance reduce the number of false-negative results due to the intake of exogenous pseudo-endogenous steroids with an isotopic signature close to the endogenous ones. However, using IRMS in an indirect longitudinal screening procedure would represent a change of paradigm and current thresholds may result in a higher risk of false positive testing from a statistical standpoint. Nevertheless, although this paradigm change complicates its implementation, the application of an IRMS approach as an additional screening tool through the development of an “isotopic module” could be a particularly promising complement to the detection of steroidal doping (28).

## Investigation of new anti-doping strategies

In the search for new biomarkers, omics strategies have also been considered (32). Designed to investigate biomarkers at a cellular level, this approach was suggested in an anti-doping approach. If *transcriptomic* and *proteomic* investigations have shown their interest (33), a *metabolomic* approach has recently demonstrated an interesting perspective (34). In this context, a recent study identified a panel of metabolites following autologous blood transfusion (35). Also applied for the screening of growth hormone (36) and testosterone misuse (37), individualized reference ranges seem to be the most promising approach (34). Nevertheless, the difficulty of setting reference values due to differences between sports disciplines (38) or various confounding factors such as intense cardiovascular effort (39) or nutritional supplements (40) constitute important challenges for field implementation. Therefore, despite some limitations, an explorative metabolomics approach looking for longitudinal profiling should be further investigated (41).

Through a microscopic approach, the morphology of red blood cells (RBC) could also be an innovative perspective in the future of blood doping detection, especially for blood transfusion. If the homologous blood transfusions (HBT) are detectable by flow cytofluorimetry-based method (42, 43), autologous blood transfusions are currently only trackable through indirect biomarkers (33). It is known that some properties of RBCs will be altered during storage (44), especially to membrane modification inducing a reduction of the deformability (45). Based on a recent study, it seems besides that deformability alteration is observed generally but also in the different subpopulations of RBC maturation (46). In this way, changes in the expression of CD 55 and CD 59 surface RBC proteins and variation in cell size have been observed (47), demonstrating the relevance of biomarkers related to blood aging and storage. More recently, a recent study investigated circulating RBC extracellular vesicles after transfusion, showing an increase of this biomarker in the hours following reinfusion, thus providing additional evidence in case of suspicious hematological profile (48). Therefore, a morphological approach to RBC membrane alterations could complement the ABP in the ABT identification and needs to be investigated.

## Screening new forms of doping

Longitudinal biomarkers sensitive to other types of doping may also be another axis of research in the development of the ABP. Being under development for more than a decade by the World Anti-Doping Agency, an endocrine module will be introduced to tackle doping and particularly to address the use of growth hormones in an ABP approach (49). Several biomarkers have hence been investigated in the screening of different growth hormones (GH), in particular procollagen type III N-terminal peptide (P-III-NP), insulin growth factor-I (IGF-I), and the GH-2000 age discriminant score resulting from these two biomarkers (50–52). The stability of these markers confirms their relevance in a longitudinal approach (52), although a large intra-individual variability seems to be observed in women (53), complicating longitudinal follow-up. Showing very encouraging results when applied to the ABP model despite short detection windows (49), these outcomes strengthen the hypothesis for the future implementation of the endocrine module to complete the ABP screening spectrum. A similar approach seems to be the only option to detect also doping by other growth factors, that are presently not detectable by other analytical approaches.

## Application of artificial intelligence

Key in today's data processing, the application of artificial intelligence (AI) in anti-doping has been investigated for several years and should be the subject of further research. By developing software gathering various types of information (e.g., physical performance or hematological data), an innovative strategy was initially developed to improve the target testing by emphasizing abnormal patterns (54). Since then, several approaches seeking to investigate multiple machine learning algorithms to identify general (55) or specific doping practices (56) have been investigated. These projects have confirmed the large potential of machine learning in anti-doping, leading the way for more elaborate design (56). Following a similar approach, performance monitoring has been suggested as an alternative to biological matrices (57). Using performance data such as competitive results (58, 59) or on critical power (60), an “athlete performance passport” has been suggested for building individualized career performance trajectories (61). Therefore, these models could contribute to trace abnormal performances and completing biological investigations in a field where AI will undoubtedly be part of future development.

## Conclusion

The development of new biomarkers is required for the later development of the ABP. Several fields of investigation are currently being pursued, in which AI will certainly play an essential role. Nevertheless, to provide specific and robust analysis, these new markers need to be studied extensively to determine the natural variability, especially in a population of elite athletes with increased influencing factors. Overall, all these resources could grant a step forward in the target testing and support anti-doping authorities in obtaining the right sample, at the right time, from the right athlete.

## Data availability statement

The original contributions presented in the study are included in the article/supplementary material, further inquiries can be directed to the corresponding author.

## Author contributions

BK and RF conceptualized the article. BK drafted the manuscript. All authors contributed to revising critically the manuscript and approved the final submitted version.

## Funding

Open access funding was provided by the University of Lausanne.

## Conflict of interest

The authors declare that the research was conducted in the absence of any commercial or financial relationships that could be construed as a potential conflict of interest.

## Publisher's note

All claims expressed in this article are solely those of the authors and do not necessarily represent those of their affiliated organizations, or those of the publisher, the editors and the reviewers. Any product that may be evaluated in this article, or claim that may be made by its manufacturer, is not guaranteed or endorsed by the publisher.
