# Erratum: Future opportunities for the athlete biological passport

**DOI:** 10.3389/fspor.2023.1173479

**Published:** 2023-03-17

**Authors:** Frontiers Production Office

**Affiliations:** Frontiers Media SA, Lausanne, Switzerland

**Keywords:** anti-doping, athlete biological passport, blood, urine, serum, biomarkers

An Erratum on Future opportunities for the athlete biological passport By Krumm B, Botrè F, Saugy JJ and Faiss R. (2022) Front. Sports Act. Living 4:986875. doi: 10.3389/fspor.2022.986875

An omission to the funding section of the original article was made in error. The following sentence has been added: “Open access funding was provided by the University of Lausanne.”

The original version of this article has been updated.

